# Nanoscale light element identification using machine learning aided STEM-EDS

**DOI:** 10.1038/s41598-020-70674-y

**Published:** 2020-08-13

**Authors:** Hong-Kyu Kim, Heon-Young Ha, Jee-Hwan Bae, Min Kyung Cho, Juyoung Kim, Jeongwoo Han, Jin-Yoo Suh, Gyeung-Ho Kim, Tae-Ho Lee, Jae Hoon Jang, Dongwon Chun

**Affiliations:** 1 Advanced Analysis Center, Korea Institute of Science and Technology, Seoul, 02792 Republic of Korea; 2 Ferrous Alloy Department, Korea Institute of Materials Science, Changwon, 51508 Republic of Korea; 3 Center for Energy Materials Research, Korea Institute of Science and Technology, Seoul, 02792 Republic of Korea

**Keywords:** Materials science, Nanoscience and technology

## Abstract

Light element identification is necessary in materials research to obtain detailed insight into various material properties. However, reported techniques, such as scanning transmission electron microscopy (STEM)-energy dispersive X-ray spectroscopy (EDS) have inadequate detection limits, which impairs identification. In this study, we achieved light element identification with nanoscale spatial resolution in a multi-component metal alloy through unsupervised machine learning algorithms of singular value decomposition (SVD) and independent component analysis (ICA). Improvement of the signal-to-noise ratio (SNR) in the STEM-EDS spectrum images was achieved by combining SVD and ICA, leading to the identification of a nanoscale N-depleted region that was not observed in as-measured STEM-EDS. Additionally, the formation of the nanoscale N-depleted region was validated using STEM–electron energy loss spectroscopy and multicomponent diffusional transformation simulation. The enhancement of SNR in STEM-EDS spectrum images by machine learning algorithms can provide an efficient, economical chemical analysis method to identify light elements at the nanoscale.

## Introduction

In a multi-component material, light elements determine the physical, chemical, mechanical, and electrical properties of the material; hence, alloying with light elements can be exploited for many applications. For example, the microstructure and phase stability in ferrous alloys are strongly dependent on the addition of a small amount of C and/or N (~ 1 wt%), which in turn dramatically changes their mechanical properties and corrosion resistance^1–5^. In addition, the distribution/concentration of light elements at the nanometer scale substantially affects the phase formation, which determines the performance of the material^6,7^. Therefore, analytical characterization techniques, strengthened by both a robust detection limit and nanometer spatial resolution, are required for researching and manufacturing materials with enhanced properties.

Analytical techniques such as scanning transmission electron microscopy (STEM)-electron energy loss spectroscopy (EELS) and 3D atom-probe tomography (3D-APT) have been widely used to characterize the chemical composition or a phase structure of materials due to their excellent detection limits (0.005–0.1 at%^8–10^ and 0.001 at%^11,12^, respectively) and spatial resolutions (0.1 nm^13,14^ and 0.2–0.4 nm^15–18^, respectively). In spite of these strengths, these techniques have some drawbacks. For example, large background EELS signals that stem from multiple scattering appear in the tails of the zero-loss peaks, resulting in a reduction in sensitivity^19^. Consequently, chemical composition results are substantially affected by the thickness of samples when using STEM-EELS. In addition, the wider usage of 3D-APT in nanoscale characterization is limited owing to the necessity of using small analytical volumes (~ 10 × 10 × 100 nm)^20,21^, the difficulty of sample preparation, and the production of local magnification artifacts caused by evaporation field-induced compositional variations^16,22^.

In contrast, STEM–energy dispersive X-ray spectroscopy (EDS) allows a detection limit as small as 0.05 wt%^23^ with nanometer spatial resolution (< 2 nm)^24,25^ and adequate efficiency of both time and cost for chemical quantification. However, the detection limits of light elements are insufficient, since less characteristic X-ray signals are generated by light elements owing to the lower number of orbiting electrons. This results in a smaller sample signal compared to the noise signal. This lower signal-to-noise ratio (SNR) restricts light element identification by STEM-EDS. Furthermore, it is difficult to detect a light element when alloyed with heavy element(s), because its characteristic X-ray energies are likely to be overlapped by the low-energy L and M peaks of the heavy element^26^.

Recently, machine learning (ML) algorithms have been applied for the classification^27–35^ and noise reduction^36–40^ of images or spectra obtained from electron microscopy. These algorithms point to a possible congregation of different signals that represent independent information but are physically correlated with each other. This is achieved using statistical algorithms that perform the dimensionality reduction without human contribution, such as singular value decomposition (SVD) and independent component analysis (ICA). Therefore, the noise signals can be distinguished from the elemental signals using these unsupervised ML algorithms, and consequently, they can be removed. Eventually, the sensitivity can be enhanced by noise-reduction of the initial data. The SVD and ICA algorithms have been applied to EDS spectrum images (SIs) of multi-component systems with nanoscale spatial resolution^29–31^, which enhanced the sensitivity of chemical composition and distribution measurements.

Herein, we present a simple, efficient, and economical analytical method for nanoscale light element characterization using STEM-EDS SIs with the aid of ML algorithms. High-nitrogen stainless steel (HNS) was used as the target alloy. This alloy has been used in a wide range of applications, from medical materials to the ship and oil industries. It is an interesting material owing to its high fatigue strength, tolerance for fracture at low and high temperatures, cold working hardening, and high wear resistance, among other features^1–3^. These characteristics originate from the high N content (> 0.4 wt%), which acts as a strong austenite stabilizer and an interstitial solid solution strengthener^2–7,41,42^, and also improves the corrosion resistance of stainless steel when in solid solution^42^. Despite these advantages, the lack of thermal stability of HNS hinders its use. When aged at 600–950 °C, the Cr and N in HNS tend to form Cr_2_N precipitates^43–50^, which reduce the mechanical properties such as tensile strength, fracture toughness, and fatigue life^46–52^, as well as the corrosion resistance^50,53,54^.

It is generally accepted that the precipitation of Cr_2_N induces the formation of Cr- and N-depleted zones around the precipitate^44,55^. Since these elements are responsible for the physico-chemical properties of stainless steels, investigating these Cr- and N-depleted zones is essential. However, various reports^44,55^ have shown that the Cr- and N-depleted regions are too narrow for substantial composition changes to be detected. Additionally, the low SNR makes it difficult to identify N depletion, although this can be overcome by enhanced detection limits and spatial resolutions. On the other hand, electrochemical methods have been employed to indirectly estimate the extent of Cr depletion, both at the grain boundary and around Cr-related precipitates, through evaluation of the intergranular corrosion behavior^43,44,56^. Additionally, direct observation of Cr-depleted regions around the Cr_2_N precipitates using techniques such as scanning electron microscopy (SEM)-EDS^47,57^, electron probe micro-analysis (EPMA)^58^, transmission electron microscopy (TEM)-EDS^59^, and STEM-EDS^60^ have been reported. However, even these techniques do not have the detection limit or SNR required for clear analysis of the N depletion.

In this study, we investigated the correlation between the element distribution around the Cr_2_N precipitate and the aging time of HNS using STEM-EDS SIs with improved SNRs via ML algorithms. Using this technique, we performed an effective and direct observation of N distribution around the Cr_2_N precipitate in HNS. The elemental spectrum was compared to spectra treated with SVD and ICA algorithms to examine whether these algorithms are effective in reducing the noise signals. Additionally, the verification process was conducted using EELS data and a multicomponent diffusional transformation simulation.

## Results and discussion

**Figure 1:**
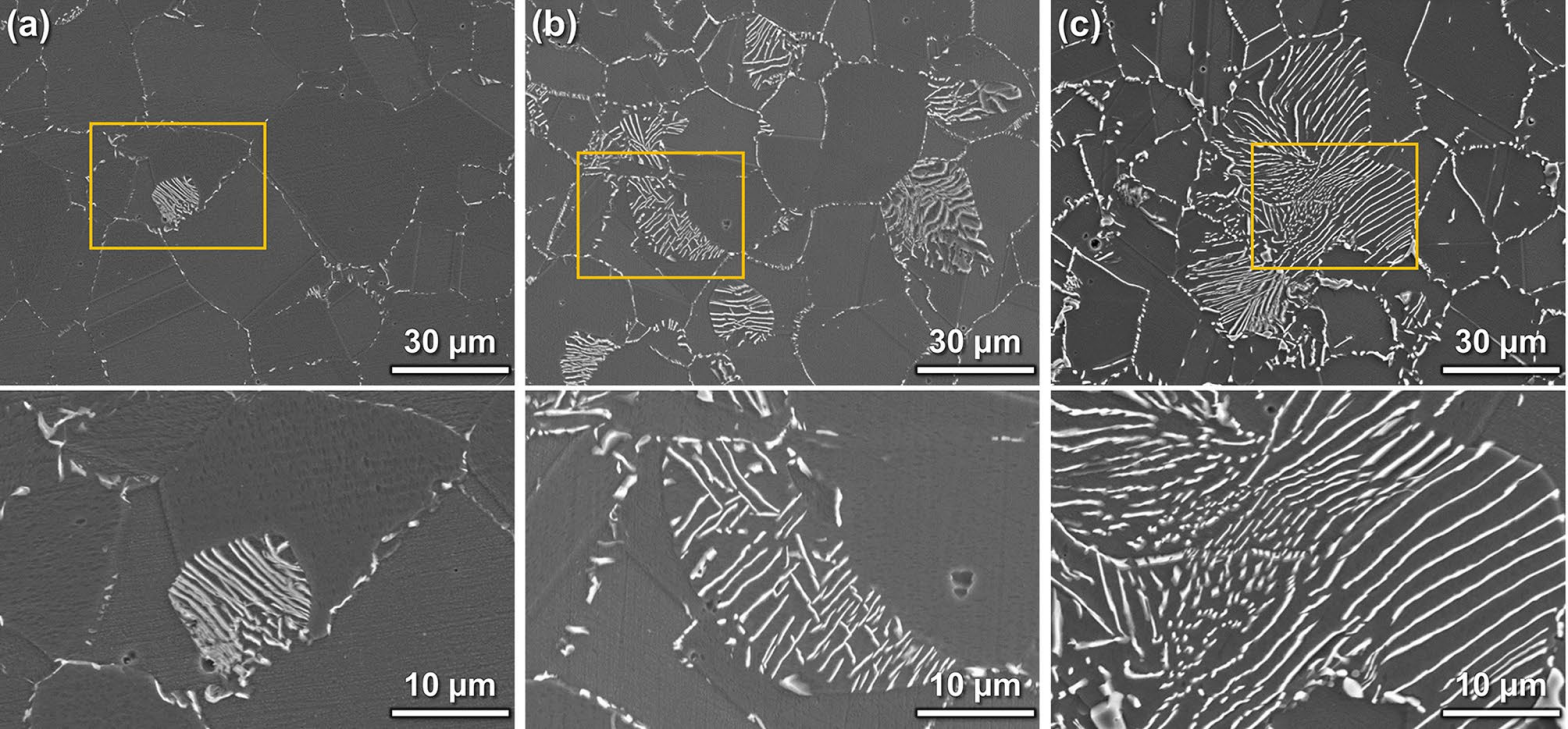
Scanning electron microscopy (SEM) images (top) and higher magnified images (bottom) of yellow box marked in each SEM image of P900NMo alloy specimens showing morphologies of Cr_2_N precipitates grown by aging at 900 °C in high-nitrogen stainless steel (HNS) for different aging times: (**a**) 10^3^ s, (**b**) 10^4^ s, and (**d**) 10^5^ s.

Figure 1 shows SEM images of the microstructures of HNS specimens aged at 900 °C for 10^3^, 10^4^, and 10^5^ s. Trigonal Cr_2_N precipitates^61–63^ were observed in the micrographs as bright white regions at the grain boundaries and within the grain with a lamellar structure. In the specimen aged for 10^3^ s (Fig. 1a), a cellular type of Cr_2_N began to form within the grains, and the volume fraction of cellular Cr_2_N increased with the aging time (Fig. 1b,c). The precipitate embryos grow by consuming other embryos or constituent elements around them, resulting in the formation of a region depleted of specific elements around the precipitate. However, the depletion zone of light elements such as N is not easily detected by conventional STEM-EDS technology because the SNR is too low. We attempted to overcome this detection limit by reducing the noise signals using unsupervised machine learning algorithms. First, the elemental distribution around the Cr_2_N precipitate was investigated using STEM-EDS. Then, the noise signals in the spectral images were reduced by combining several unsupervised machine learning algorithms. Finally, by comparing the noise-reduced STEM-EDS, EELS, and simulation results, the depletion zone of light elements was confirmed.

**Figure 2:**
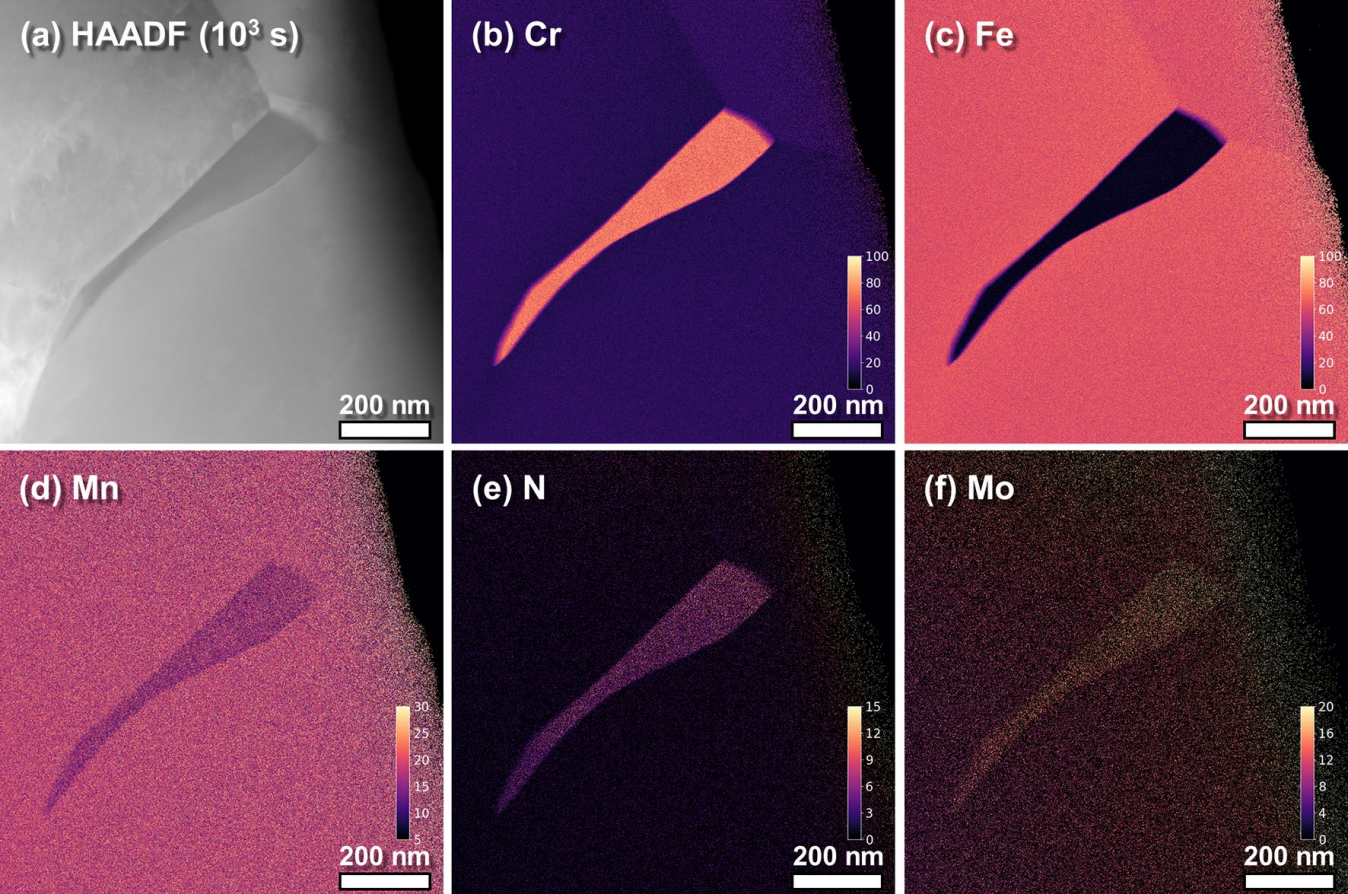
High-angle annular dark-field imaging (HAADF)-scanning transmission electron microscopy (STEM) and energy dispersive X-ray spectroscopy (EDS) elemental mapping images of the Cr_2_N precipitate in the specimen aged at 900 °C for 10^3^ s: (**a**) HAADF-STEM image, (**b**) Cr EDS map, (**c**) N EDS map, (**d**) Fe EDS map, (**e**) Mn EDS map, and (**f**) Mo EDS map.

**Figure 3:**
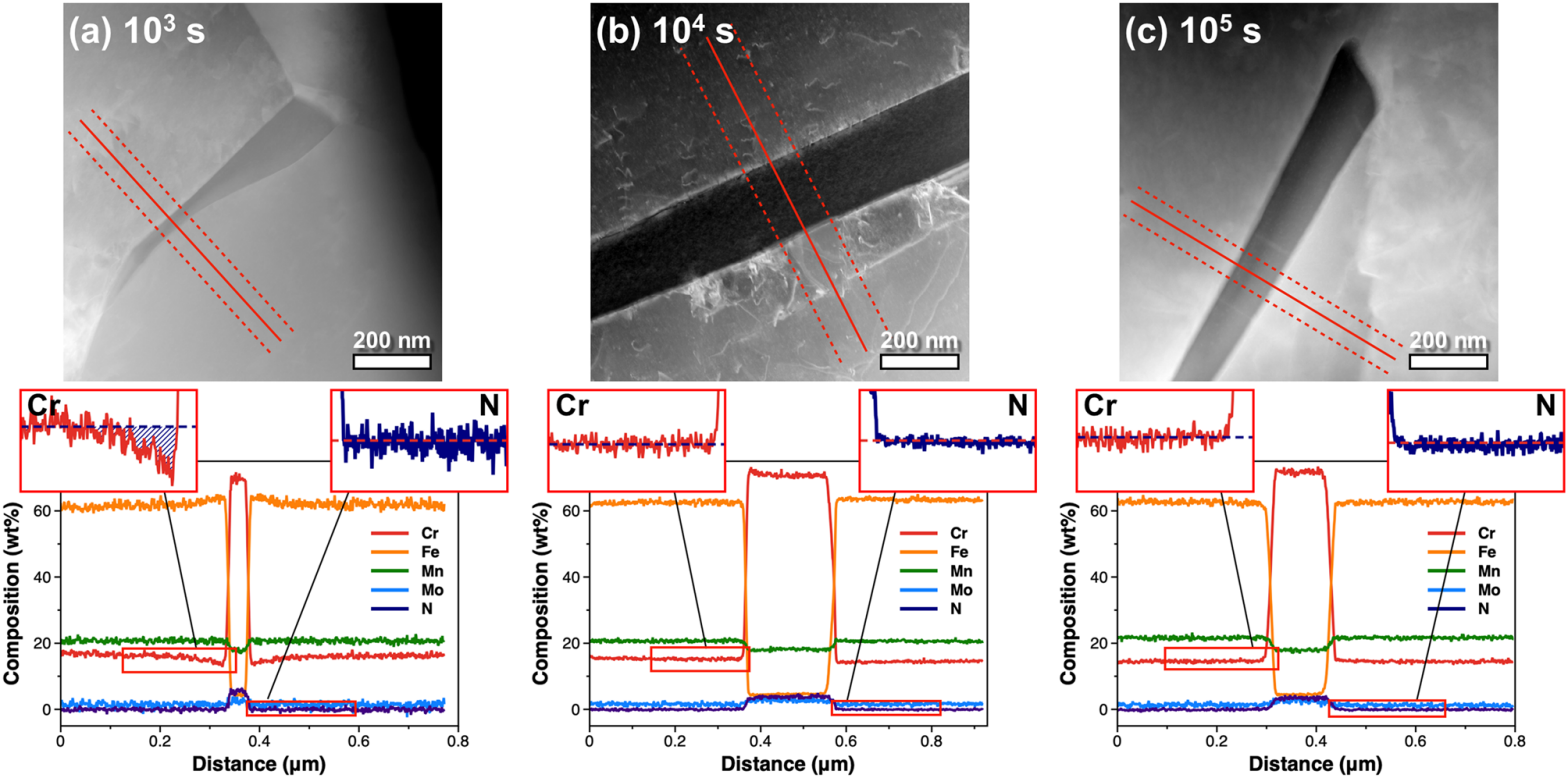
High-angle annular dark-field imaging (HAADF)-scanning transmission electron microscopy (STEM) micrographs of Cr_2_N precipitates (top) and composition line profiles for Cr, Fe, Mn, Mo, and N (bottom) along the red lines marked in the HAADF-STEM images of specimens aged at 900 °C for different aging times: (**a**) 10^3^ s, (**b**) 10^4^ s, and (**c**) 10^5^ s. The composition line profiles of Cr and N are magnified and displayed as insets, showing whether the depletion region of Cr and N is observed.

Figure 2 shows the high-angle annular dark-field imaging (HAADF)-STEM and EDS mapping images of a typical precipitate in the HNS sample aged at 900 °C for 10^3^ s. The precipitates had a cellular morphology and width of 100–150 nm. The EDS maps show that the main components of the precipitate and matrix were Cr and Fe, respectively (see Fig. 2b,c, respectively). There was minimal Fe in the precipitate, while Mn, N, and Mo were all present (Fig. 2d–f). The concentration of Fe atoms in the precipitate was less than 5% of that in the matrix (for details, see Fig. 3a), which suggests that the precipitate does not overlap with the matrix alloy, or is placed on a very thin matrix layer that can be considered negligible. Thus, the characteristic X-ray signals of Mn and Mo within the precipitate, as shown in Fig. 2e,f, respectively, do not result from the matrix alloy but from the precipitate itself. The concentration of Mn atoms within the precipitate was smaller than that within the matrix, while the concentration of Mo atoms was greater. The morphology and width of the precipitates and the respective distribution of each element (including each element’s concentration) were similar to that in the samples aged for 10^4^ and 10^5^ s (see Supplementary Figs. S1 and S2 online).

For more quantitative analysis, the EDS concentrations of each element were profiled along the red lines in Fig. 3a–c. The concentrations of each element in the matrix and within the precipitate were similar for the samples aged for 10^3^, 10^4^, and 10^5^ s; the Cr, Fe, Mn, Mo, and N concentrations in the matrix were approximately 16, 63, 21, 2, and 0.2 wt%, respectively, and those with in the precipitate were 71, 4, 18, 2.5, and 4.3 wt%, respectively. The difference in matrix composition between the nominal composition and EDS results stems from the uncertainty of electrons scattered into a detector^30^. Within the precipitate, the Cr and N concentrations were approximately 71 and 4 wt%, respectively, regardless of the aging time. However, the Cr and N concentrations around the precipitate, i.e., in the depletion region, were dependent on the aging time. In the sample aged for 10^3^ s, a Cr-depleted zone was observed around the precipitate, with a minimum Cr concentration of 13 wt% (adjacent to the precipitate; see the left inset of the composition line profile in Fig. 3a for details), while no such reduction in Cr concentration was observed for the samples aged for 10^4^ or 10^5^ s (see the left insets in Fig. 3b,c, respectively). This difference is likely to result from the diffusion of Cr atoms from the matrix to the region around the precipitates in the samples aged for 10^4^ and 10^5^ s. Nevertheless, this does not explain the absence of an N-depleted region in the sample aged for 10^3^ s (right inset in Fig. 3a), because the interstitial diffusion of N atoms is faster than that of the other substitutional elements. Considering the low concentration of N in HNS, it is conceivable that the flat concentration profile resulted from difficulties in distinguishing N signals and noise.

**Figure 4:**
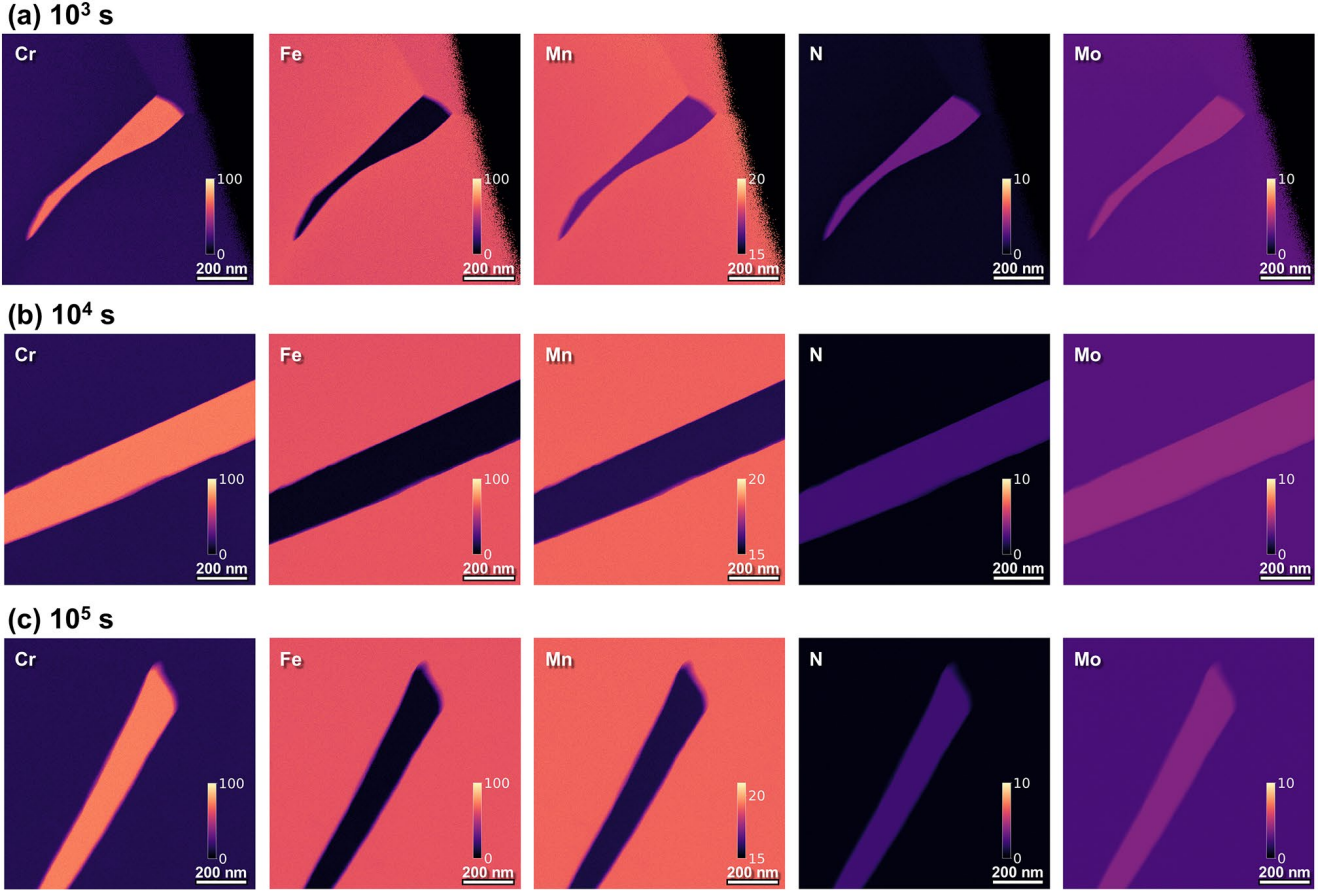
Images with enhanced resolution due to reduced noise signals. Energy dispersive X-ray spectroscopy (EDS) mapping images reconstructed using only a few principal components of the samples aged at 900 °C for different aging times: (**a**) 10^3^ s, (**b**) 10^4^ s, and (**c**) 10^5^ s.

To investigate the presence of the N-depleted region around the Cr_2_N precipitate, the noise signals of the SIs were reduced using the SVD and ICA algorithms. The EDS maps were reconstructed using only a few principal components following decomposition using the SVD and ICA^64–66^ and selected based on a knee-point detecting algorithm^67^ (for details, see Supplementary Figs. S3–S5 online). Figure 4 shows the reconstructed EDS maps of the samples aged for 10^3^, 10^4^, and 10^5^ s. The Cr, Fe, and Mn elemental maps do not differ much from the original maps. This suggests that the principal components selected based on the knee-point algorithm provide enough information to represent most of the variation of the characteristic X-ray signals, while also reproducing the elemental configuration. However, where the element had a relatively small concentration, such as N and Mo, the SNR of the elemental maps was considerably enhanced by the noise reduction process (for details, see Supplementary Fig. S6 online). The magnitude of characteristic X-ray signals from the majority elements (Cr, Fe, and Mn) is sufficiently higher than that of the noise signals; therefore, reduction of the noise signals has a negligible effect on the original spectral data. The opposite was observed for N and Mo, where the original SNR was much lower.

**Figure 5:**
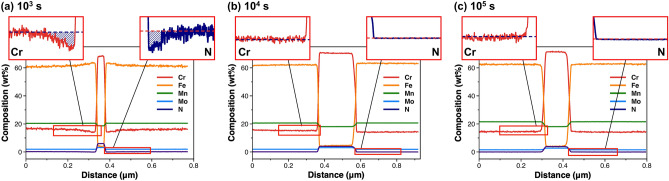
Energy dispersive X-ray spectroscopy (EDS) line profiles of each element for the samples aged at 900 °C for different aging times: (**a**) 10^3^ s, (**b**) 10^4^ s, and (**c**) 10^5^ s. The composition of each element is profiled along the same red lines marked in the high-angle annular dark-field imaging (HAADF)-scanning transmission electron microscopy (STEM) images in Fig. 3.

To confirm whether the remarkable SNR enhancement in the N and Mo spectral data would reveal the presence of the N-depleted region, we re-examined the compositional line profiles of the precipitate and surrounding area. In order to make a fair comparison with the line profiles in Fig. 3, the concentrations of each element were profiled at exactly the same position and width, as shown in Fig. 5. (Line profiles integrated at other positions are presented in Supplementary Figs. S7–S9 online.) The resulting compositional line profiles of all elements except N were equivalent to those from the original spectral images. However, an N-depleted region was clearly revealed in the sample aged for 10^3^ s following noise reduction, as shown in the right inset in Fig. 5a. The width of the Cr- and N-depleted regions were almost identical at 70–100 nm, indicating that the diffusions of Cr and N atoms were considerably correlated. Additionally, the minimum Cr concentration in the depletion region was 13 wt% (adjacent to the precipitate), which coincides with the result obtained from the line profile in Fig. 3a, while the minimum N concentration was 0.01 wt% (adjacent to the precipitate). Compared to the Cr and N concentrations in the matrix of approximately 16 and 0.2 wt%, respectively, the depletion region was deficient in Cr by ≤ 3 wt% and in N by ≤ 0.19 wt%. Cr- and N-depleted regions were not observed in the samples aged for 10^4^ and 10^5^ s. Thus, an aging time of 10^4^ s seems to be sufficient for Cr and N atoms to diffuse into the depletion region around the Cr_2_N precipitates. These observations were confirmed by summing the EDS signals in an area for each of the regions of interest, i.e., the matrix, depletion region, and precipitate (see Supplementary Fig. S10 online for details).

It is almost impossible to quantify the detection limit in EDS images because of the discontinuity of the characteristic X-ray signals; therefore, we evaluated the degree of enhancement for the detection limit of EDS images by calculating the SNR (Table 1). The EDS mapping images of abundant elements like Cr, Fe, and Mn were slightly or negligibly enhanced, but those of sparse elements like Mo and N were drastically improved on the SNR (or the detection limit), with improvements of 470% and 44%, respectively.

**Table 1:** Signal-to-noise ratios (SNRs) calculated for energy dispersive X-ray spectroscopy (EDS) elemental mapping images before and after noise reduction (NR).

Element	Cr	Fe	Mn	Mo	N
SNR (before NR)	1.29	1.95	1.95	0.43	1.03
SNR (after NR)	1.34	1.99	2.38	2.45	1.48

**Figure 6:**
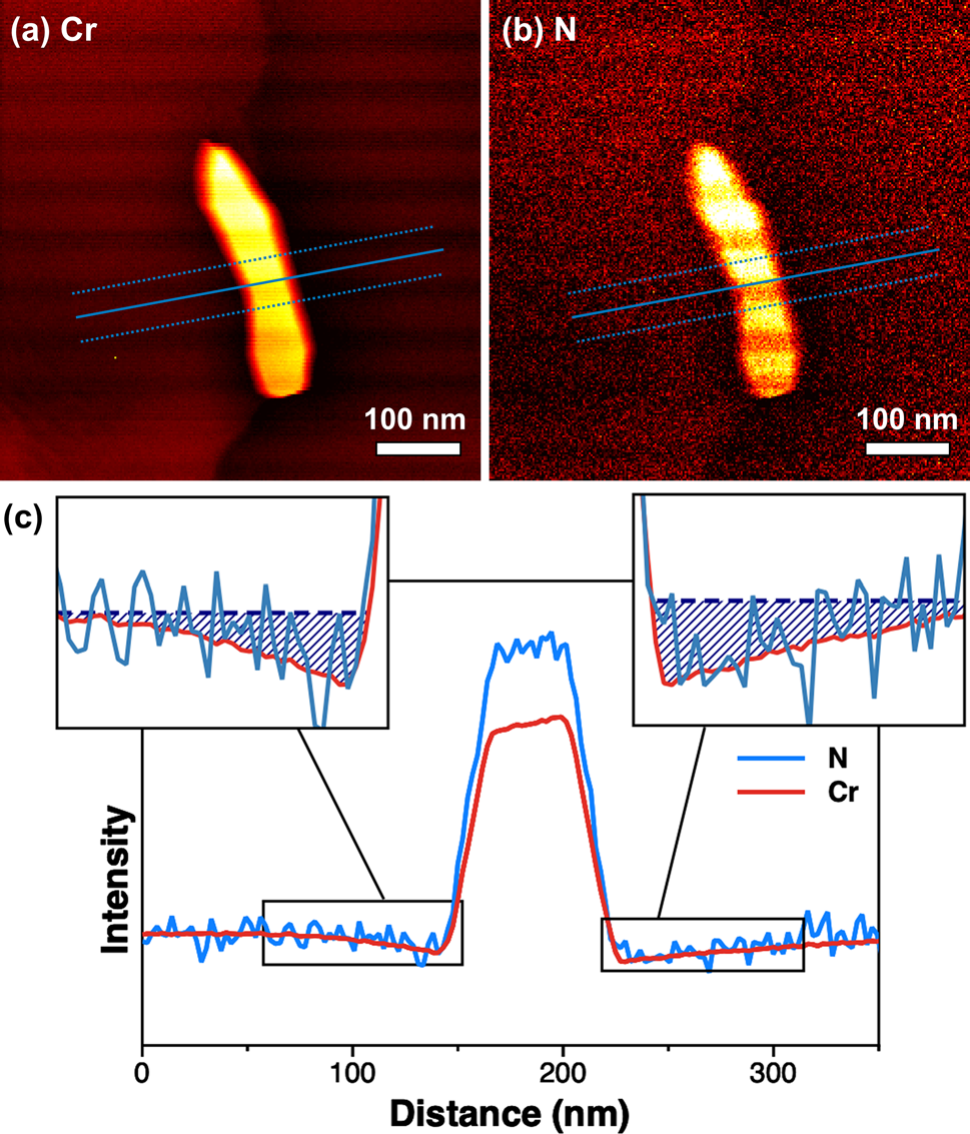
Electron energy loss spectroscopy (EELS) elemental maps of (**a**) Cr and (**b**) N constituting the Cr_2_N precipitate, and (**c**) their line profiles. The line profiles are obtained along the blue lines indicated in the EELS mapping images, and regions in black boxes are magnified as insets. The line profile of N was multiplied by an appropriate value to allow comparison with that of Cr.

We performed EELS analysis of a Cr_2_N precipitate to validate the EDS results. The EELS elemental maps of Cr and N (Fig. 6a,b, respectively) provide information about the Cr- and N-depleted regions, with these regions being clearly recognized in the line profiles (see Fig. 6c and Supplementary Fig. S11 online). To compare the Cr and N EELS line profiles, the intensity of the N profile was adjusted to that of the Cr profile by multiplication with an appropriate value. Interestingly, the depletion regions of Cr and N coincided precisely, as shown in Fig. 6c. Both regions had a width of approximately 70–100 nm, which is the same as the width of the depletion regions obtained from the EDS results (Fig. 5a). This confirms that the noise reduction achieved by the proposed technique successfully increases the SNR without loss of information from the original signals. In general, the efficiency of EELS analysis in terms of both time and cost is inferior to that of EDS analysis. In addition, to eliminate the effect of sample thickness on the EELS signals, the plural scattering signals must be removed from raw EELS data, with the risk of distorting the spectra. From this perspective, EDS analysis with machine learning algorithms is more effective than EELS for detecting light elements.

**Figure 7:**
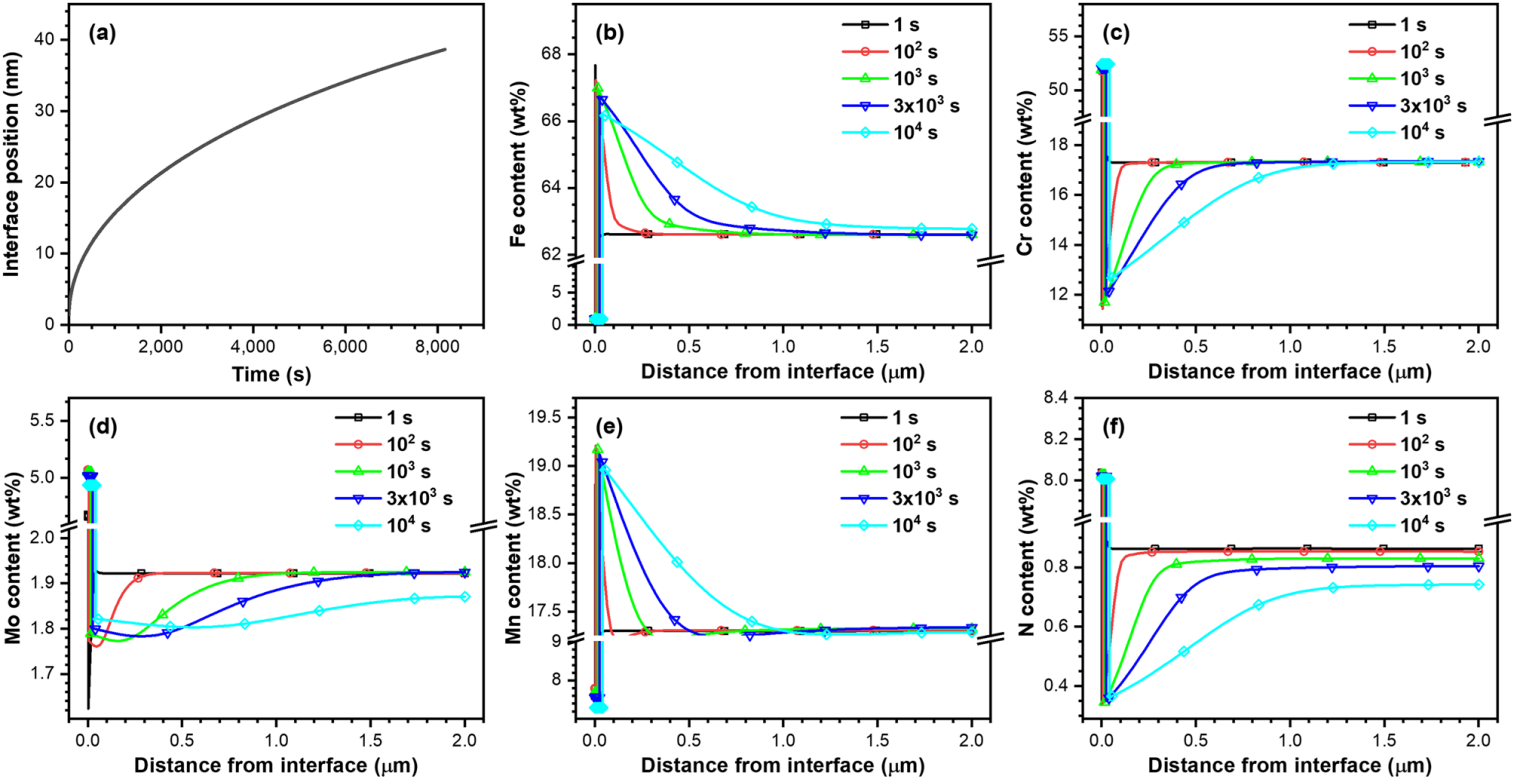
Results of a multicomponent diffusional transformation simulation showing Cr_2_N precipitate growth. (**a**) Precipitate size as a function of temperature, and alloying element contents profile for (**b**) Fe, (**c**) Cr, (**d**) Mo, (**e**) Mn, and (**f**) N.

To better understand the reason behind the similar widths of the N- and Cr-depleted regions, we explored the diffusional dynamics of each element, i.e., Fe, Cr, Mo, Mn, and N, using numerical simulations to solve the diffusion equation. The simulation results are summarized in Fig. 7. The austenite and Cr_2_N phases are both thermodynamically stable at 900 °C. Hence, the direction in which the interface moves is related to the equilibrium fractions of Cr_2_N and austenite. Figure 7b–f show the concentration profile changes for each element in the whole system at different times. The element concentrations change abruptly at the interface of the two phases. Fe, Cr, and N have relatively large concentration gradients at the submicron scale when the heat treatment is less than 10^3^ s. This means that the probability of observing Cr- and N-depleted regions in the sample aged for 10^3^ s is higher than that in the samples aged for longer than 10^3^ s. Additionally, the simulation results coincided with the EDS and EELS experiments. It is important to note that, over time, the gradient of N is similar to that of Cr (Fig. 7c,f, respectively), which could be attributed to the chemical potential effect caused by the Cr concentration gradient in the matrix. This happens despite the diffusion coefficient of N being approximately five orders of magnitude higher than that of other substitutional elements. These diffusional dynamics induced by the chemical potential effect force the width of the N-depleted region to correspond with the Cr-depleted region.

The compositional profile of alloying elements near the precipitate is essential for understanding the evolution of the precipitate. However, it is difficult to measure the profile of light elements such as N. Machine learning algorithms, such as SVD and ICA, can successfully reveal not only Cr deficiency but also N deficiency, which is regarded as the primary reason for degradation of various mechanical and corrosive properties, around the Cr_2_N precipitates that form in HNS. The physico-chemical properties of steel alloys depend on the distribution of precipitates. Therefore, an advanced analysis of the distribution of precipitates is important for the design of high-performance steels. The precise detection and analysis technique suggested in this study can be utilized in a comprehensive interpretation of the evolution kinetics of nanometer-sized precipitates containing light elements, and consequently can result in the design of an optimum thermal treatment process.

## Conclusions

The combination of two unsupervised machine learning algorithms, i.e., SVD and ICA, successfully reduces the noise signals in EDS images and therefore increases the SNR of images. The N-depleted region around the Cr_2_N precipitate, which was concealed by noise signals in the original EDS data, was revealed using this technique. This is significant owing to the difficulties of noise separation and removal through normal signal processing methods. The Cr- and N-depleted regions were only observed in samples aged for 10^3^ s when using our proposed method. The widths of the Cr- and N-depleted regions were equal, ranging from 70 to 100 nm. This consistency was validated using EELS. Simulations provided further evidence for the diffusional dynamics that explain how N, with lighter and faster diffusion, follows the depletion behavior of Cr. Both the simulation and EELS results support our method as a feasible and useful way of increasing the SNR in spectral images of different natures, including EDS and EELS. The work reported in this study can be viewed as a potential way of identifying light elements, such as N, from EDS experiments, in a more efficient way than that of EELS experiments. Other popular decomposition methods, such as non-negative matrix factorization (NMF), also provide the same results suggested in this work (see Supplementary Fig. S12 online). Thus, it is valuable to explore and compare different multivariate analysis algorithms for identifying light elements, which we will explore in future work.

## Methods

### Sample preparation

The HNS was a commercial P900NMo alloy (manufactured by VSG, Essen, Germany) with a composition of Fe_bal._–17.94Cr–18.60Mn–2.09Mo–0.89N–0.04C (in wt%), which is a modified version of P900 (DIN 1.3816) with higher Mo and N concentrations. Specimens (12 × 10 × 4 mm) were cropped from the hot-rolled plate, encapsulated in an evacuated quartz tube, solution-treated at 1,150 °C for 30 min, and water-quenched. The resulting specimens were isothermally aged at 900 °C for 10^3^, 10^4^, and 10^5^ s under Ar, followed by water-quenching. At this aging temperature, Cr_2_N formation is facilitated while the formation of other precipitates is retarded (e.g., σ phase)^50,61,68,69^. After isothermal aging, the microstructure for each specimen was analyzed using SEM (JSM-7100F, JEOL, Japan). For this analysis, the aged specimens were mechanically ground with SiC abrasive papers to 2,400 grit, mechanically polished using a diamond suspension with a particle size of 1 μm, and chemically etched in a glyceregia reagent (10 mL nitric acid, 20 mL hydrochloric acid, and 30 mL glycerin) at 25 ± 1 °C for 1–2 min followed by rinsing with water and drying in air.

### Electron microscopy analysis

To investigate the elemental configuration changes and aging time of the depletion region using STEM-EDS, samples with different aging times were prepared using a focused ion beam (FIB; Helios NanoLab 600, FEI, US) lift-off milling technique. The Cr_2_N precipitates were observed using TEM (Talos F200X, FEI, US) at an accelerating voltage of 200 kV (Schottky X-FEG gun) and equipped with a Super-X EDS system comprising four windowless silicon drift detectors (SDDs) in STEM mode with a probe current of ~ 0.7 nA. To guarantee a high enough SNR, the EDS mapping data was collected through a spectrum imaging form for 60 min with a 20 ms/pixel dwell time. This large dwell time also allows the Bremsstrahlung background subtraction based on a simple and widely used two-window method. The windows for each element (Cr, Fe, Mn, N, and Mo) are denoted in Supplementary Fig. S13 (online). After the background removal, we quantified the composition of each element in the HNS samples using this spectrum imaging data based on the conventional Cliff–Lorimer method with *k*-factors provided by the manufacturer (Bruker).

EELS signals were obtained using a Quantum 966 (Gatan, USA) spectrometer attached to a Cs-corrected microscope (Titan 80-300, FEI, Netherlands), with an energy resolution of 0.8 eV for 0.01 eV/channel energy dispersion. The convergence semi-angle for the incident beam was 36 mrad, with an EELS collection semi-angle of ~ 50 mrad.

### Noise reduction using machine learning algorithms

To reduce the noise signals in the STEM-EDS images, principal component analysis (PCA) and ICA, which are machine learning algorithms for dimensional reduction, were performed using the HyperSpy package^70^, written in Python. The noise-reduced EDS mapping images were obtained by the following three steps: (1) decomposition of the multivariate X-ray signals using the SVD algorithm; (2) independent component analysis; and (3) reconstruction of de-noised EDS maps. For the PCA, the spectral energy information of each pixel in the spectral images obtained by STEM-EDS was decomposed using the SVD technique. Spectral image with spatial dimensions of 1,024 × 1,024 and an energy dimension of 4,096 was decomposed by computing the SVD as follows:

$$M=U\Sigma {V}^{T}$$

where *M* is a 1024^2^ × 4,096 spectral image matrix, *U* is a 1024^2^ × 1024^2^ factor matrix vector, Σ is a diagonal 1024^2^ × 4,096 eigenvalue matrix with non-negative values, and *V*^*T*^ is the conjugate transpose with a 4,096 × 4,096 loading matrix vector. In terms of matrix factorization, the factor and loading matrix can be expressed as follows:

$$M{M}^{T}=\left(U\Sigma {V}^{T}\right){\left(U\Sigma {V}^{T}\right)}^{T}=U\left(\Sigma {\Sigma }^{T}\right){\mathrm{U}}^{T},$$

$${M}^{T}M={\left(U\Sigma {V}^{T}\right)}^{T}\left(U\Sigma {V}^{T}\right)=V\left({\Sigma }^{T}\Sigma \right){\mathrm{V}}^{T}.$$

Then, in view of eigenvalue decomposition, *U* and *V*, which are eigenvectors of *MM*^*T*^ and *M*^*T*^*M*, respectively, can be calculated by solving the eigenvalue characteristic equations:

$$\left(M{M}^{T}-\lambda I\right)x=0,$$

$$\left({M}^{T}M-\lambda I\right)x=0,$$

where *λ* represents the eigenvalues and *x* the eigenvectors, which can be transformed to the *U* and *V* matrices. Consequently, the principal components were derived with Σ*V*^*T*^. Since the noise signal is subject to the Poisson distribution due to the uncertainty of electrons, the Poissonian noise normalization method was adapted into all of the decomposing processes. Then, the ICA, known as blind source separation^64–66^, was performed using the FastICA algorithm^71^ embedded in the HyperSpy package to enhance the physical correlation between the principal components. As the factor matrix was derived from the SVD calculations, FastICA was used to find a maximum of the *w*^*T*^*U* non-Gaussianity, where *w* is the weight vector. To do this, an initial weight vector was randomly selected, and the vector matrix was recalculated until it converged, as shown in the following equation:

$${w}^{+}=E\left\{Ug\left({w}^{T}U\right)\right\}-E\left\{{g}^{^{\prime}}\left({w}^{T}U\right)\right\}w,$$

$$w= \frac{{w}^{+}}{\Vert {w}^{+}\Vert },$$

where *E*{*x*} is the variance of the *x* matrix and *g*(*x*) is the derivative of the non-quadratic function. Finally, the independent components were obtained by multiplying *w* and *U*. The independent components with high eigenvalues that represent most variances were used for the reconstruction of the de-noised EDS mapping images. This was conducted using a PCA scree plot and a knee-point detecting approach^67^ (for details, see the PCA scree plots, signals, and maps of the independent components in Supplementary Figs. S3–S5 online).

To evaluate the SNR of the spectral images, the coefficient reciprocal of the variation calculation method^72^ was adopted. Briefly, for each element constituting the Cr_2_N precipitate, appropriate ranges of energy in the spectral images were summed. Then, given images containing intensities of the elemental signals, the SNR was calculated as follows:

$$\mathrm{SNR}=\frac{\mu }{\sigma },$$

where *µ* is the expected value of the intensities of signals in the image, and *σ* is the standard deviation of the noise. This method has been widely used for SNR quantification in the field of image and signal processing^73–75^.

### Multicomponent diffusional transformation simulation

Cr_2_N precipitate growth was simulated using multicomponent diffusional transformation (DICTRA module, Version 2018a, Thermo-Calc. Software AB, Sweden)^76^ software using thermodynamics (TCFE7.0) and mobility (MobFe2) databases^77–79^. This software obtains a numerical solution of the diffusion equation at the local equilibrium in the phase interface. Assuming there is no difference in the chemical potential at the interface between the matrix (austenite) and precipitate (Cr_2_N), the alloying element concentration at the interface can be evaluated from the thermodynamic equilibrium. The rate of phase transformation was controlled by the rate of the incoming or outgoing diffusional flux of elements. The software can simulate the growth process of the Cr_2_N precipitate in austenite assuming diffusion-controlled growth by solving equations of thermodynamic phase equilibrium, flux balance, and diffusion. The conservation of mass leads to the following flux balance conditions at the moving interface between the austenite matrix and Cr_2_N precipitate:

$$V\left({C}_{k}^{\mathrm{austenite}}-{C}_{k}^{{\mathrm{Cr}}_{2}\mathrm{N}}\right)={J}_{k}^{\mathrm{austenite}}-{J}_{k}^{{\mathrm{Cr}}_{2}\mathrm{N}}, \left(k = \mathrm{1,2}, \dots , n\right),$$

where *V* is the interface migration rate, ${C}_{k}^{\mathrm{austenite}}$ and ${C}_{k}^{{\mathrm{Cr}}_{2}\mathrm{N}}$ are the concentration of species *k* in austenite and Cr_2_N close to the interface, respectively, and ${J}_{k}^{\mathrm{austenite}}$ and ${J}_{k}^{{\mathrm{Cr}}_{2}\mathrm{N}}$ are the diffusion flux in austenite and Cr_2_N, respectively. These can be expressed according to Fick’s first law of diffusion^77^:

$${J}_{k}=-\sum_{j=1}^{n-1}{D}_{kj}^{n}\nabla {C}_{j} ,$$

where *n* is the number of elements, ${D}_{kj}^{n}$ is the diffusion coefficient of the matrix, and *∇C*_*j*_ is the concentration gradient for element *j*.

The growth of the Cr_2_N precipitate was simulated using the moving boundary model of the DICTRA software. It was assumed that the austenite and Cr_2_N phases are separated by a planar boundary, and that thermodynamic equilibrium exists locally at the interface. Initial conditions were set where 1 nm of Cr_2_N is bound by a 2 μm layer of austenite. The initial Cr_2_N composition was assumed to be the same as the thermodynamic equilibrium results at 900 °C. The austenite composition was set as Fe_bal._–18Cr–18Mn–2Mo–0.9 N (wt%). The concentration was calculated for 20 uniform points within Cr_2_N and 200 uniform points within austenite. The transition of the interface and the concentration profiles at the interface were calculated for the sample aged at 900 °C for 10^4^ s.

## Supplementary information

Supplementary Information.

## Data Availability

The datasets generated during and/or analysed during the current study are not publicly available due to preparing another study and patent but are available from the corresponding author on reasonable request.
