# Supplementary material for: Nanoscale light element identification using machine learning aided STEM-EDS
Source: Sci Rep. 2020 Aug 13;10:13699. doi: 10.1038/s41598-020-70674-y (PMC7426414; doi:10.1038/s41598-020-70674-y)
Supplement: Supplementary file 1 — Supplementary Information. [file 41598_2020_70674_MOESM1_ESM.docx]

Supplementary Information

**Nanoscale Light Element Identification using Machine Learning Aided STEM-EDS**

Hong-Kyu Kim^1,†^, Heon-Young Ha^2,†^, Jee-Hwan Bae^1^, Min Kyung Cho^1^, Juyoung Kim^1^, Jeongwoo Han^1^, Jin-Yoo Suh^3^, Gyeung-Ho Kim^1^, Tae-Ho Lee^2^, Jae Hoon Jang^2,*^, Dongwon Chun^1,*^

^a^Advanced Analysis Center, Korea Institute of Science and Technology, 02792, Seoul, Republic of Korea

^b^Ferrous Alloy Department, Korea Institute of Materials Science, 51508, Changwon, Republic of Korea

^c^Center for Energy Materials Research, Korea Institute of Science and Technology, 02792, Seoul, Republic of Korea

^†^First author

*Corresponding authors: Jae Hoon Jang (E-mail: jhjang@kims.re.kr)

Dongwon Chun (E-mail: chundream98@kist.re.kr)


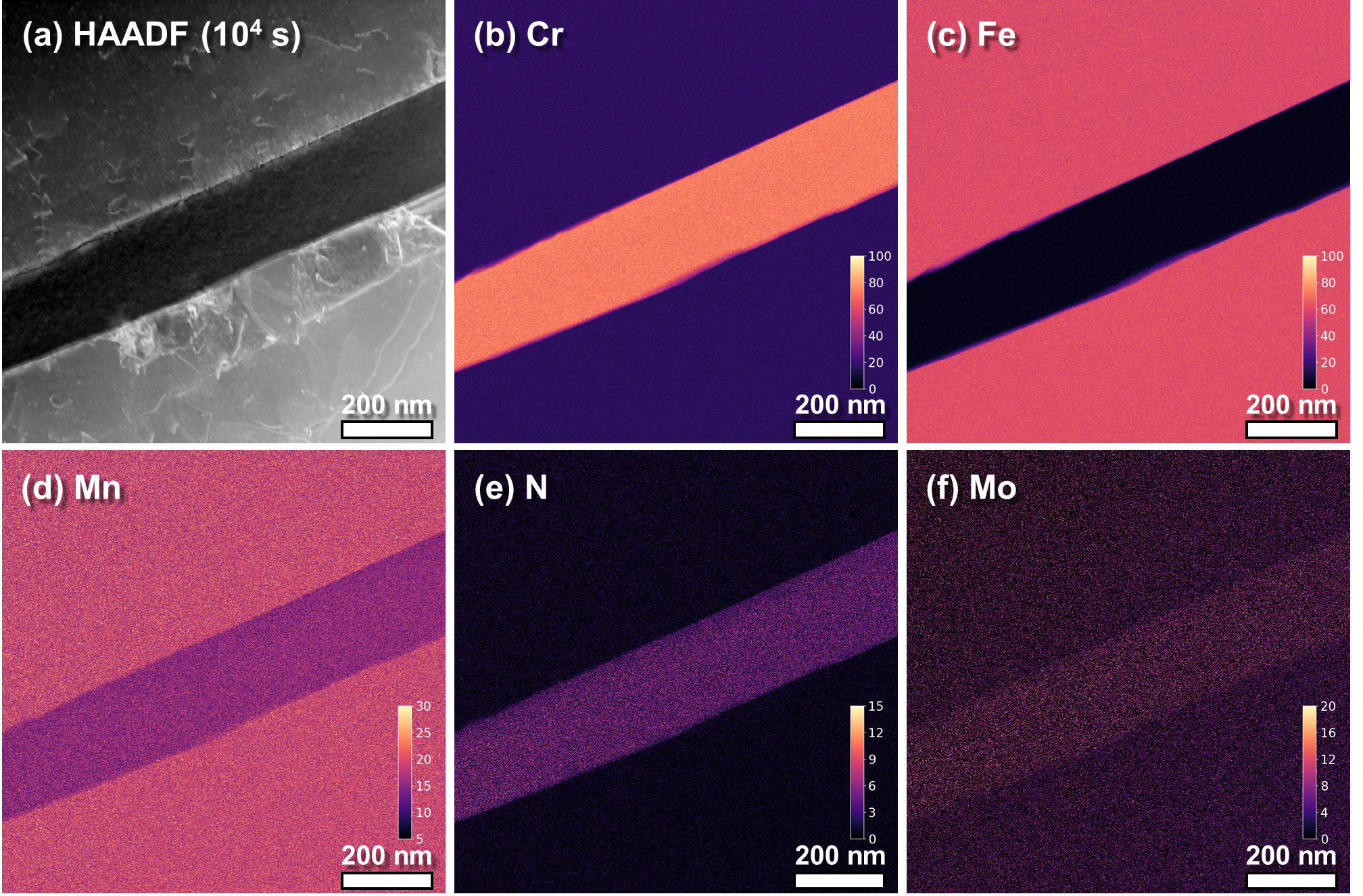


**Figure S1.** High-angle annular dark-field imaging (HAADF)-scanning transmission electron microscopy (STEM) and energy dispersive X-ray spectroscopy (EDS) elemental mapping images of a Cr_2_N precipitate in a high-nitrogen stainless steel (HNS) sample aged at 900 °C for 10^4^ s: (a) HAADF-STEM image, (b) Cr elemental map, (c) N elemental map, (d) Fe elemental map, (e) Mn elemental map, and (f) Mo elemental map.


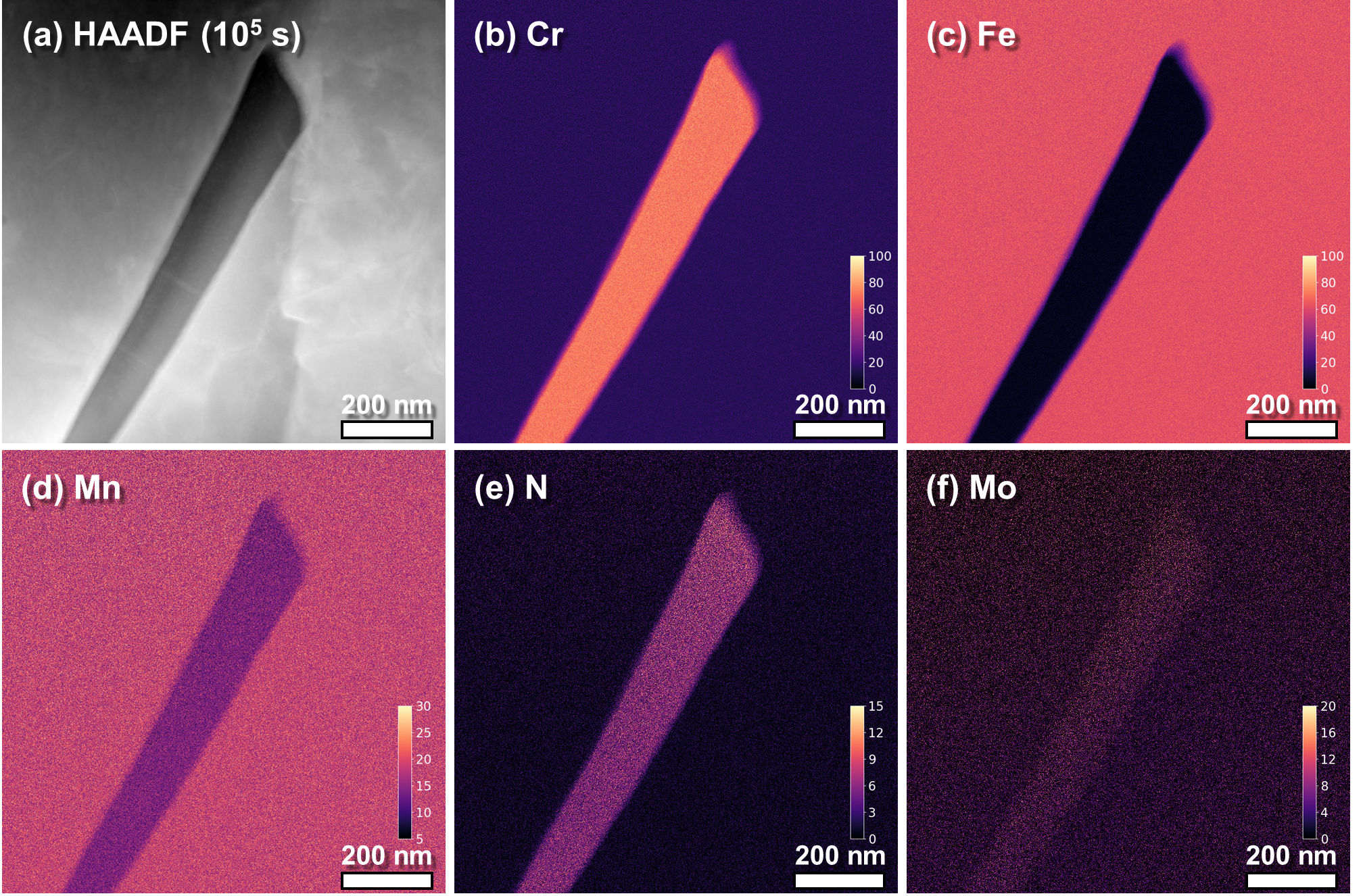


**Figure S2.** High-angle annular dark-field imaging (HAADF)-scanning transmission electron microscopy (STEM) and energy dispersive X-ray spectroscopy (EDS) elemental mapping images of the Cr_2_N precipitate in a high-nitrogen stainless steel (HNS) sample aged at 900 °C for 10^5^ s: (a) HAADF-STEM image, (b) Cr elemental map, (c) N elemental map, (d) Fe elemental map, (e) Mn elemental map, and (f) Mo elemental map.


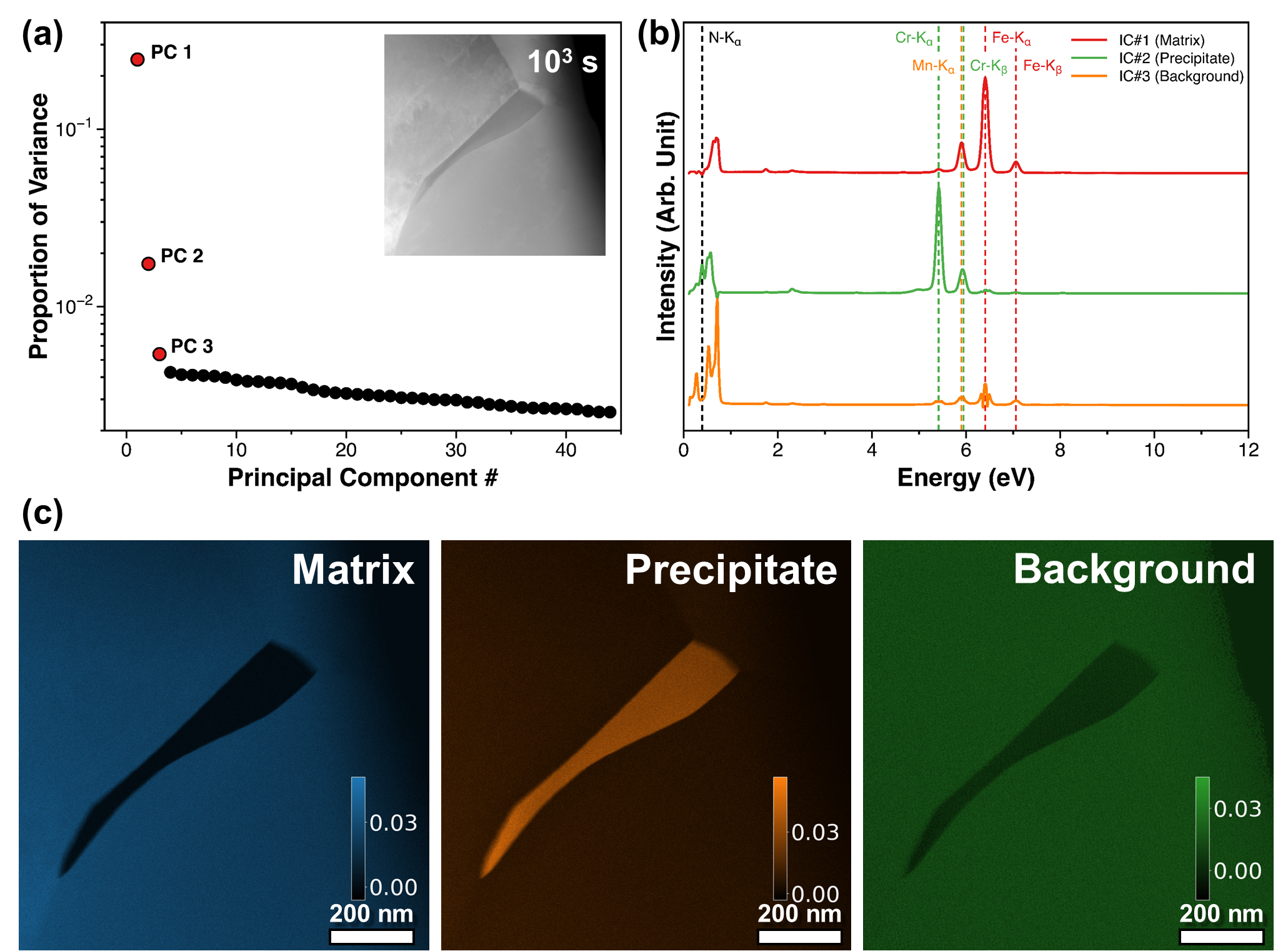


**Figure S3.** Results of the singular value decomposition (SVD) and independent component analysis (ICA) noise reduction algorithms for the energy dispersive X-ray spectroscopy (EDS) signals and elemental maps of a high-nitrogen stainless steel (HNS) sample aged for 10^3^ s: (a) principal component analysis (PCA) scree plot of first 45 components, (b) signals (factors), and (c) maps (loadings) of independent components with high variances, corresponding to the matrix, precipitate, and background.


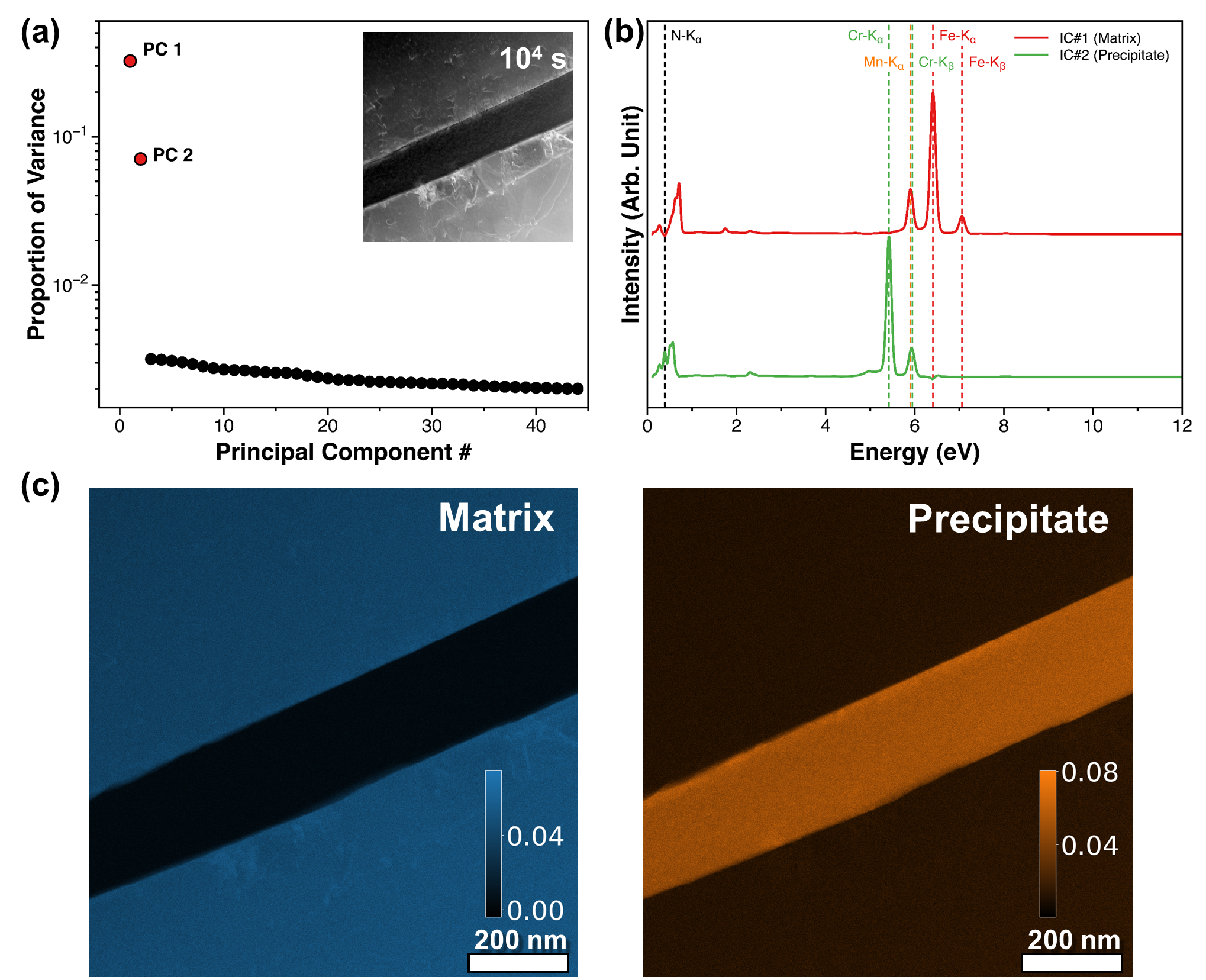


**Figure S4.** Results of the singular value decomposition (SVD) and independent component analysis (ICA) noise reduction algorithms for the energy dispersive X-ray spectroscopy (EDS) signals and elemental maps of a high-nitrogen stainless steel (HNS) sample aged for 10^4^ s: (a) principal component analysis (PCA) scree plot of the first 45 components, (b) signals (factors), and (c) maps (loadings) of the independent components with high variances, corresponding to the matrix, precipitate, and background.


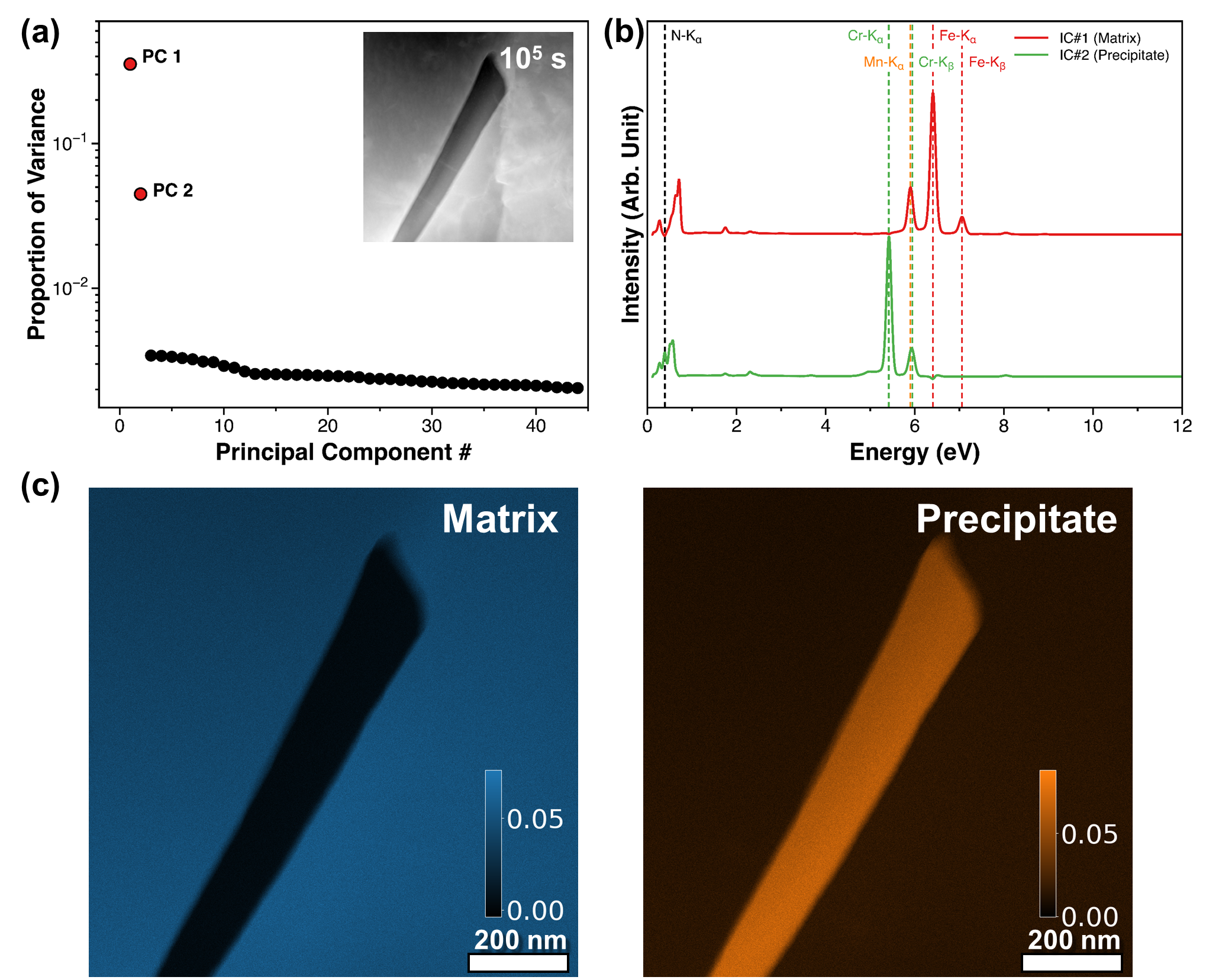


**Figure S5.** Results of the singular value decomposition (SVD) and independent component analysis (ICA) noise reduction algorithms for the energy dispersive X-ray spectroscopy (EDS) signals and elemental maps of a high-nitrogen stainless steel (HNS) sample aged for 10^5^ s: (a) principal component analysis (PCA) scree plot of the first 45 components, (b) signals (factors), and (c) maps (loadings) of the independent components with high variances, corresponding to the matrix, precipitate, and background.


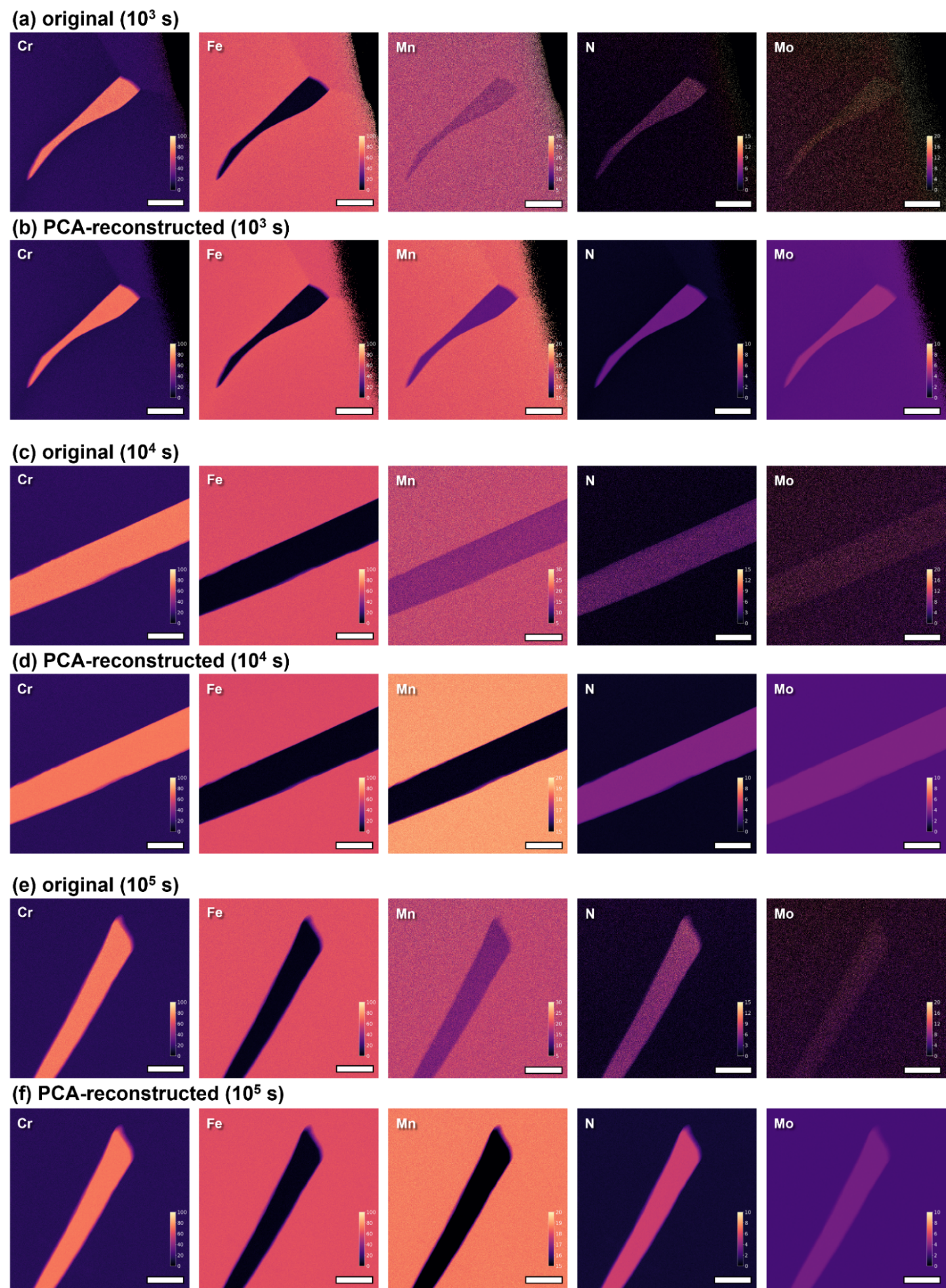


**Figure S6.** (a, c, e) Original and (b, d, f) principal/independent component analysis (PCA/ICA)-reconstructed energy dispersive X-ray spectroscopy (EDS) mapping images of high-nitrogen stainless steel (HNS) samples aged at 900 °C for (a, b) 10^3^ s, (c, d) 10^4^ s, and (e, f) 10^5^ s. The reconstruction was conducted using only a few of the independent components.


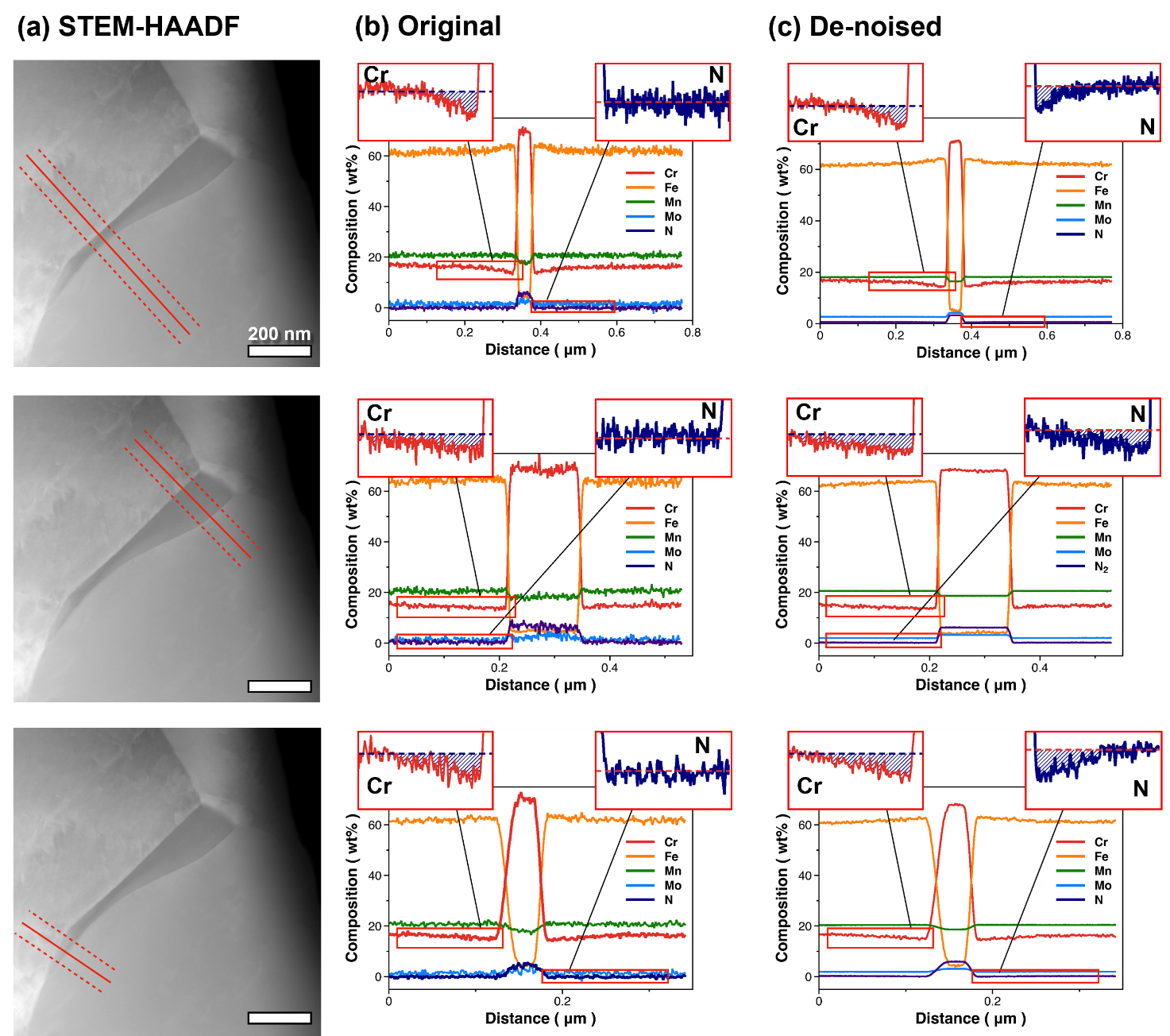


**Figure S7.** (a) High-angle annular dark-field imaging (HAADF)-scanning transmission electron microscopy (STEM) images and line profiles of (b) original and (c) de-noised energy dispersive X-ray spectroscopy (EDS) signals integrated with different positions. All images and profiles were obtained from a high-nitrogen stainless steel (HNS) sample aged for 10^3^ s.


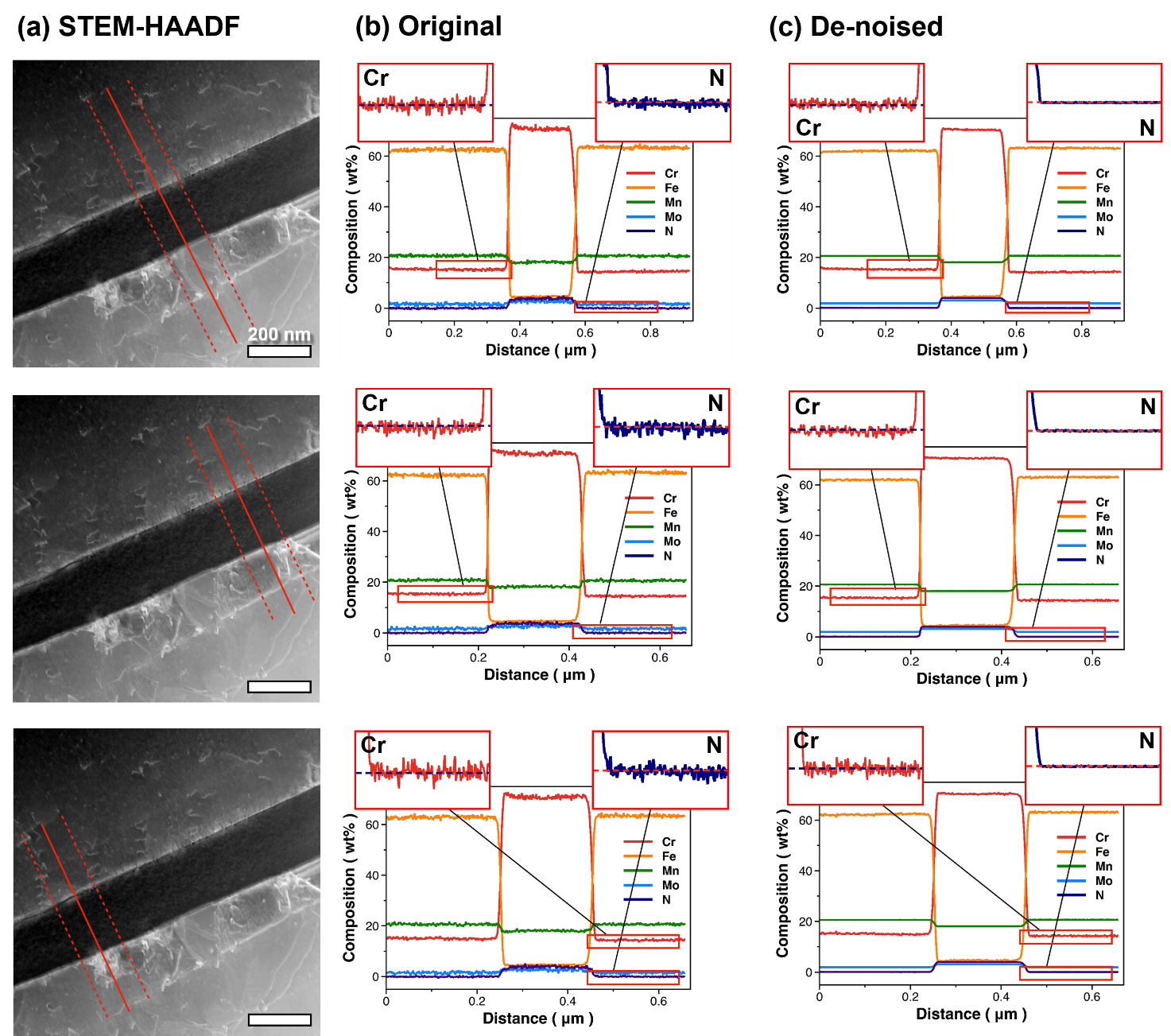


**Figure S8.** (a) High-angle annular dark-field imaging (HAADF)-scanning transmission electron microscopy (STEM) images and line profiles of (b) original and (c) de-noised energy dispersive X-ray spectroscopy (EDS) signals integrated with different positions. All images and profiles were obtained from a high-nitrogen stainless steel (HNS) sample aged for 10^4^ s.


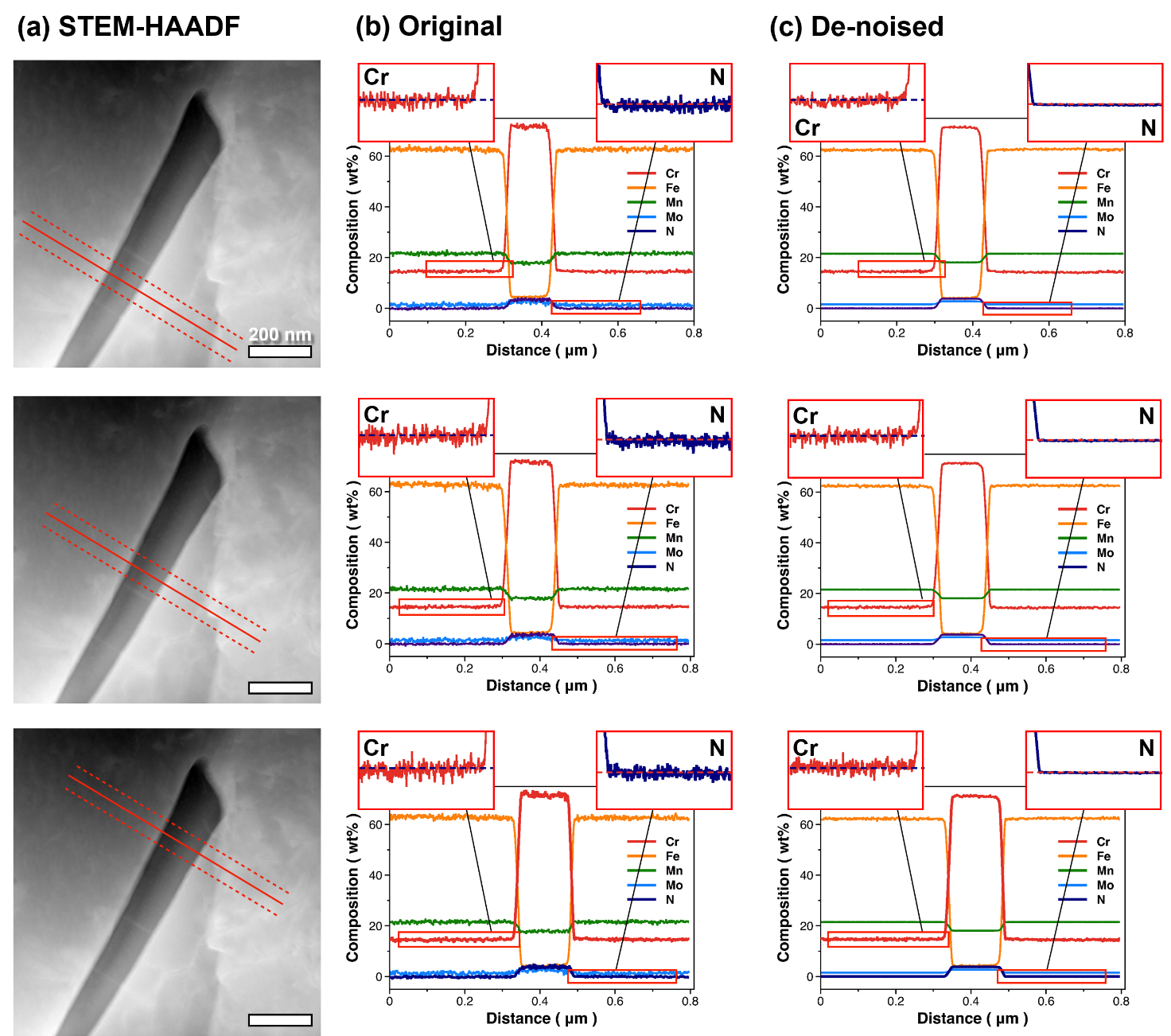


**Figure S9.** (a) High-angle annular dark-field imaging (HAADF)-scanning transmission electron microscopy (STEM) images and line profiles of (b) original and (c) de-noised energy dispersive X-ray spectroscopy (EDS) signals integrated with different positions. All images and profiles were obtained from a high-nitrogen stainless steel (HNS) sample aged for 10^5^ s.

**
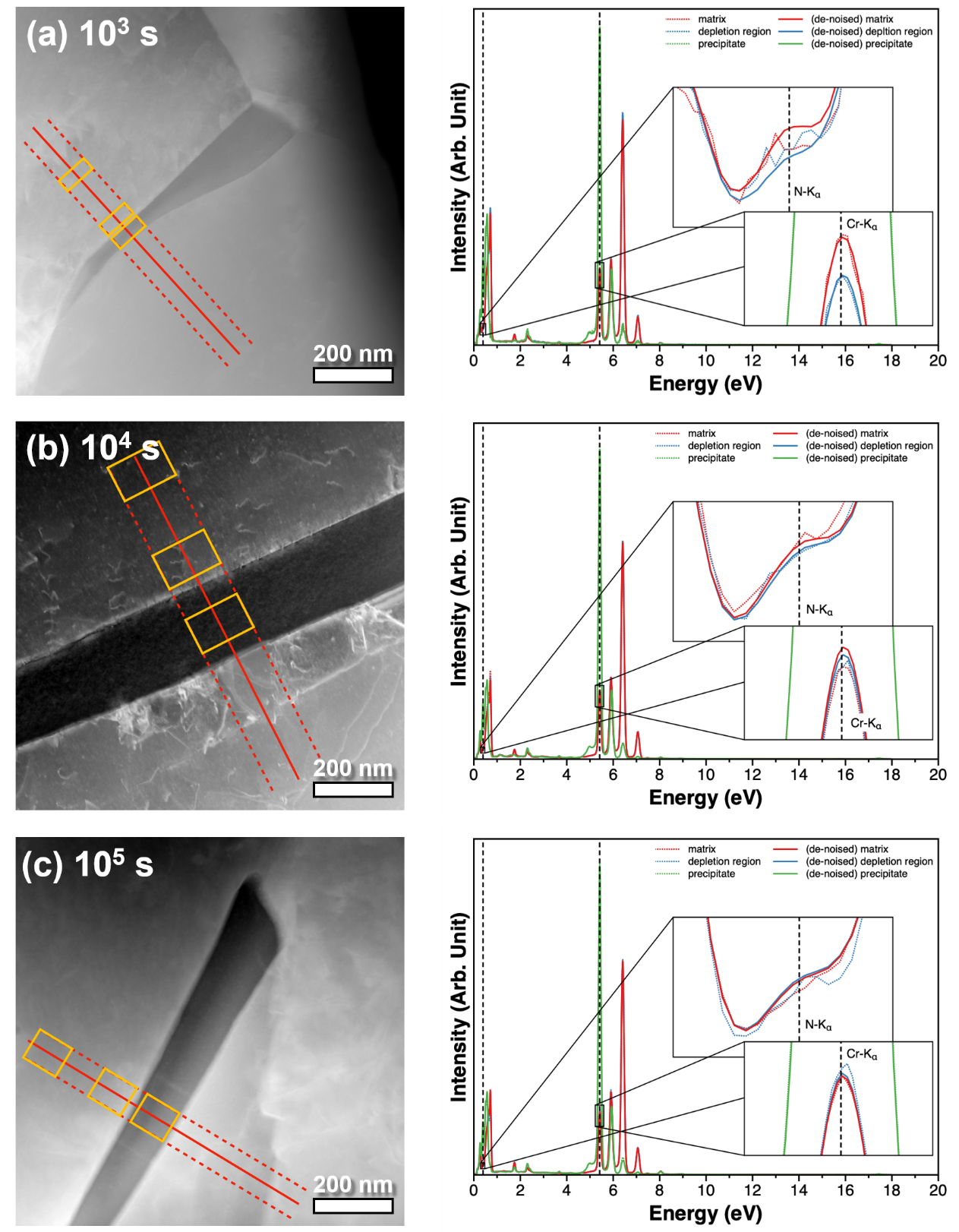
**

**Figure S10.** High-angle annular dark-field imaging (HAADF)-scanning transmission electron microscopy (STEM) images (left) of high-nitrogen stainless steel (HNS) samples aged for (a) 10^3^ s, (b) 10^4^ s, and (c) 10^5^ s and summed energy dispersive X-ray spectroscopy (EDS) signals (right) for each of the regions of interest denoted by the yellow boxes in the STEM-HAADF images: matrix, depletion region, and precipitate. The signals obtained from the de-noised images reveal Cr- and N-depleted regions in the HNS sample aged for 10^3^ s.


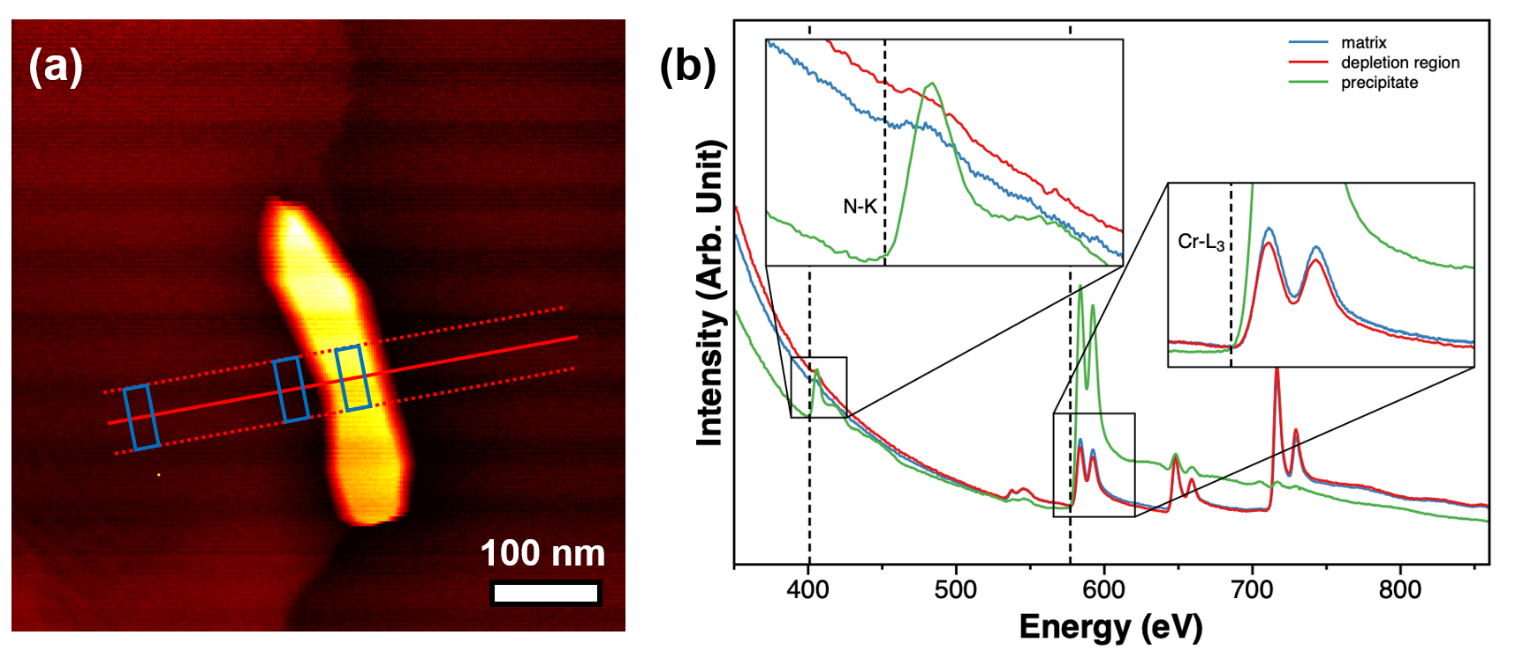


**Figure S11.** Electron energy loss spectroscopy (EELS) (a) mapping image and (b) summed signals in the matrix, depletion region, and precipitate areas (denoted by the blue boxes in (a)). The edges of Cr and N are relatively lower in the depletion region than in the matrix. For clarity, the signals around the N K- and Cr L_3_-edges are magnified and depicted as insets.


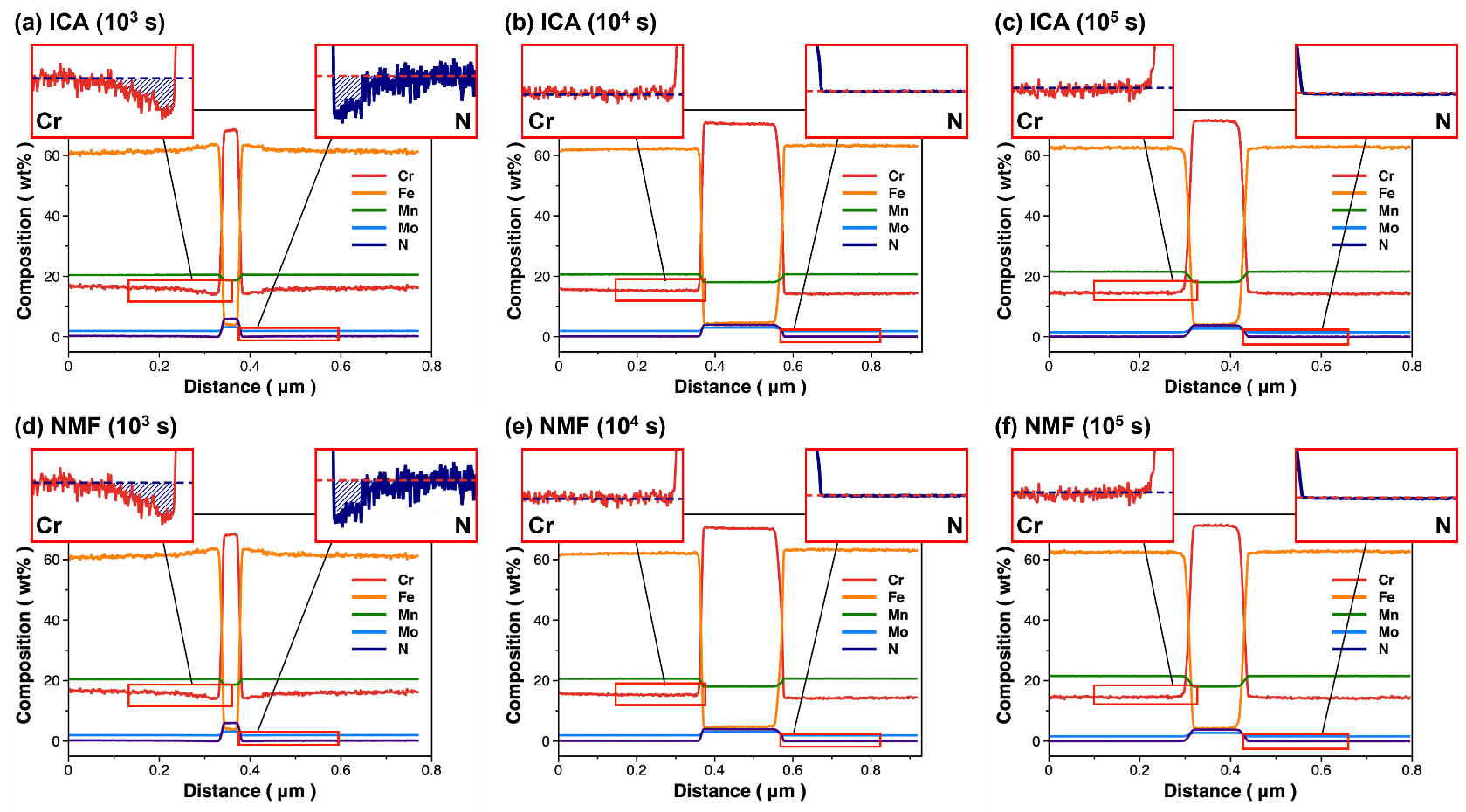


**Figure S12.** Line profiles of the energy dispersive X-ray spectroscopy (EDS) spectral images reconstructed by the (a–c) independent component analysis (ICA) and (d–f) non-negative matrix factorization (NMF) method, showing that there is no difference between the line profiles in high-nitrogen stainless steel (HNS) samples aged for same time: (a, d) 10^3^ s, (b, e) 10^4^ s, and (c, f) 10^5^ s.


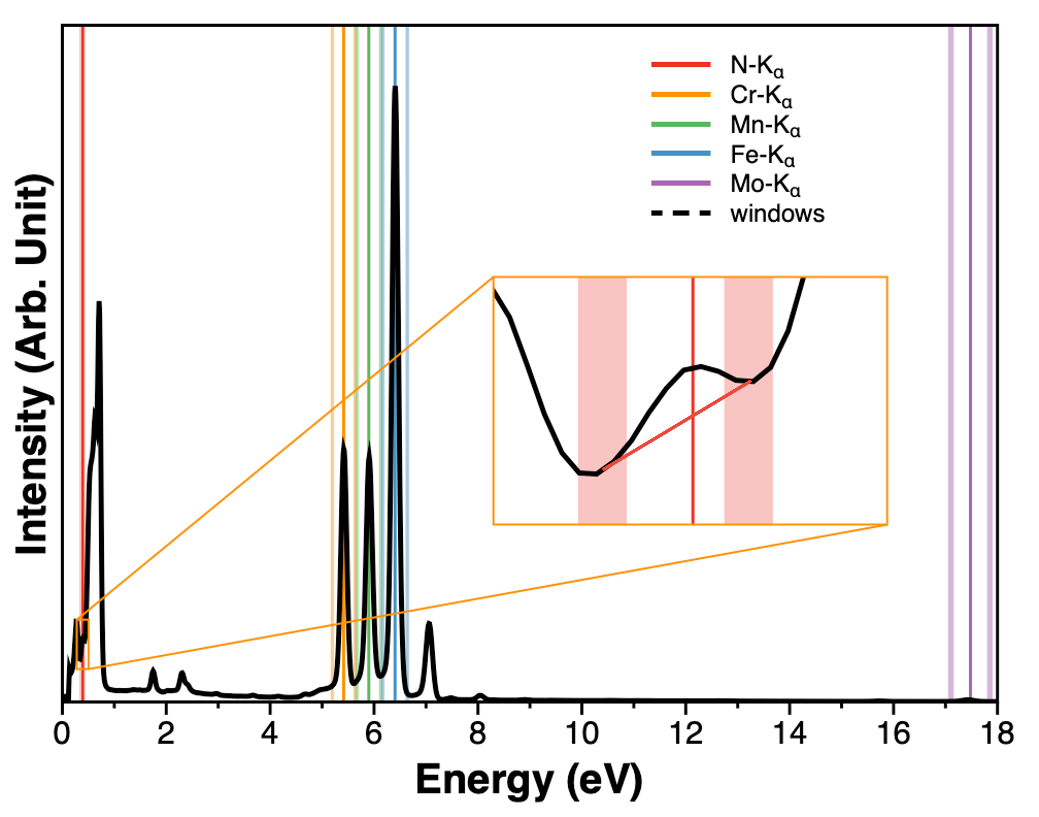


**Figure S13.** Summed X-ray spectra of a high-nitrogen stainless steel (HNS) sample aged for 10^3^ s. Kα signals of each element (N, Cr, Mn, Fe, and Mo) were used for quantification. The Kα lines are denoted as red (N), orange (Cr), green (Mn), blue (Fe), and purple (Mo) solid lines. The window of each element used for background removal are denoted with the same colors. For clarity, the region around the N Kα line (red line) is magnified in the inset.
